# Investigation of Renal Potassium Loss and Anion Gap Patterns in Diabetic Ketoacidosis: A Study From South India

**DOI:** 10.7759/cureus.106044

**Published:** 2026-03-28

**Authors:** Anjali Jayakumar, Teny Mathew, Sibin K Prasad, Sunil Mathew

**Affiliations:** 1 Department of General Medicine, Pushpagiri Institute of Medical Sciences and Research Centre, Tiruvalla, IND

**Keywords:** anion gap, diabetic ketoacidosis, hypokalemia, renal potassium loss, serum magnesium, serum potassium

## Abstract

Objective: This study aims to assess the extent and patterns of renal potassium loss and delineate anion gap profiles in patients with diabetic ketoacidosis (DKA) presenting to emergency departments in India. By characterizing these patterns, the study aims to optimize electrolyte replacement strategies and provide deeper insights into the unmeasured contributors to metabolic acidosis in DKA, thereby supporting improved clinical outcomes.

Methodology: This cross-sectional observational study was conducted over 17 months in the Emergency Department of a tertiary care hospital in Kerala to assess electrolyte disturbances in DKA. Fifty-one adult patients with confirmed DKA were enrolled. Data were collected at presentation, prior to treatment, and included clinical parameters, serum electrolytes, anion gap, and urine indices. Renal potassium wasting was evaluated using FEK and urine potassium/creatinine ratio. Laboratory analyses followed standard protocols, and statistical analysis was performed using Statistical Package for the Social Sciences version 25 (IBM Corp., Armonk, NY).

Results: Among the patients, 31 (60.8%) presented with an elevated anion gap >14 mEq/L, indicating high-anion-gap metabolic acidosis (HAGMA), with a mean of 18.19 ± 3.09 mEq/L. A total of 19 (37.3%) patients had a mildly elevated anion gap of 12-14 mEq/L, with a mean of 13.4 ± 0.54 mEq/L, while only one (2.0%) patient had a normal anion gap in the range of 10-12 mEq/L, with a value of 11.8 mEq/L. The difference in mean anion gap among these groups was statistically significant (analysis of variance: F = 39.73, p < 0.001). Renal potassium loss was evaluated in patients with hypokalemia. In mild hypokalemia cases, urine creatinine was 157 mg/dL and urine potassium was 65 mmol/L, resulting in a K⁺/creatinine ratio of 0.41 mmol/mg. In the single case of moderate hypokalemia, urine creatinine was 180 mg/dL and urine potassium was 55 mmol/L, yielding a K⁺/creatinine ratio of 0.31 mmol/mg. These findings indicate renal potassium wasting in both mild and moderate hypokalemia cases.

Conclusion: The study found that while most DKA patients had normokalemia, significant cases of hyperkalemia and renal potassium loss were present. A strong positive correlation between serum potassium and magnesium indicated interdependent regulation. Elevated anion gaps confirmed HAGMA as a dominant pattern.

## Introduction

Diabetes mellitus is a leading public health problem worldwide, and India is commonly referred to as the diabetes capital of the world, accounting for 17% of the global population [1,2]. Diabetic ketoacidosis (DKA) is a life-threatening acute metabolic emergency of diabetes. It presents with hyperglycemia, ketonemia, and anion gap metabolic acidosis. DKA affects patients mainly in the age range of 45-65 years, and it is most frequently precipitated by insulin deficiency, infections, cardiovascular or endocrine emergencies, and surgical stress [3,4]. It is frequent in individuals with type 1 diabetes and can occasionally occur in type 2 diabetes, particularly during episodes of severe physiological stress [5].

In DKA, severe insulin deficiency and increased concentrations of counterregulatory hormones, including glucagon, cortisol, catecholamines, and growth hormone, lead to metabolic derangements [6]. These hormonal changes promote lipolysis and hepatic fatty acid oxidation, leading to the excessive production of ketone bodies, particularly β-hydroxybutyrate and acetoacetate. The gradual buildup of these acids causes marked metabolic acidosis [7,8].

Despite well-established treatment protocols, electrolyte imbalances, especially potassium, continue to represent a challenge in managing these patients [9]. While serum potassium (K⁺) may initially be normal or elevated secondary to extracellular shifts, potassium is markedly deficient in the total body in DKA [10]. Insulin administration and correction of acidosis cause potassium to shift back into cells, leading to hypokalemia. In addition, osmotic diuresis in DKA results in substantial urinary potassium loss, which requires close monitoring [11].

Biochemically, the high anion gap in DKA has been historically associated with the presence of ketones [3]. This association is primarily due to the accumulation of ketone bodies, particularly β-hydroxybutyrate and acetoacetate, which are generated through enhanced lipolysis and hepatic fatty acid oxidation in the setting of insulin deficiency. These ketoacids dissociate into hydrogen ions and their corresponding anions, thereby contributing to metabolic acidosis and an elevated anion gap. However, conventional laboratory methods, such as the nitroprusside reaction, predominantly detect acetoacetate and may underestimate β-hydroxybutyrate, the predominant ketone body in DKA. Consequently, measured ketone levels may not fully explain the extent of anion gap elevation. In addition, emerging evidence suggests that other unmeasured anions, including d-lactate and various organic acids, may also contribute to the increased anion gap, reflecting a more complex biochemical basis of acidosis in DKA [12-14]. Nevertheless, the measured values for acetone and acetoacetate (by the nitroprusside reaction) do not account for the high gap response [12-14]. The primary source of gluconeogenic substrate, β-hydroxybutyrate, is overlooked and sometimes underestimated in routine examination [12]. In addition, recent data suggest that unmeasured anions, such as d-lactic acid, can play a significant role in the DKA-associated anion gap [14].

In addition to ketoacids, other unmeasured anions can contribute to the elevated anion gap in DKA. One such contributor is d-lactate, the stereoisomer of l-lactate, which is produced via the glyoxalase pathway from methylglyoxal, a by-product of glucose metabolism that accumulates under hyperglycemic conditions [15]. Elevated d-lactate levels have been reported in diabetic patients, particularly in those with DKA, and may be nearly twice as high as in healthy controls [16-18].

Although the pathophysiology of DKA is well described globally, significant gaps remain in understanding the patterns of renal potassium loss and the complete spectrum of anion contributors in Indian patients presenting to the emergency department. Most available data are derived from Western populations [7-11], which may not be directly applicable due to genetic makeup, dietary habits, environmental factors, and healthcare infrastructure differences. In India, where the prevalence of diabetes continues to rise, addressing these gaps could provide critical insights to refine electrolyte management and improve clinical outcomes in DKA.

Hence, the present study was designed to investigate the prevalence and patterns of renal potassium losses and the anion gap profile in patients with DKA in the emergency departments of an Indian setting. Insight into these patterns provides a more rational basis for electrolyte replacement, leading to more comprehensive and definitive answers regarding the unmeasured component of metabolic acidosis, ultimately helping achieve better clinical outcomes.

## Materials and methods

Study design and settings

This cross-sectional observational study was carried out in the Emergency Room of the Pushpagiri Institute of Medical Sciences and Research Centre, a tertiary care referral center in Tiruvalla, Kerala, from January 2023 to May 2024. Ethical clearance was obtained from the Institutional Ethics Committee (IEC No. PIMSRC/E1/388A/53/2022).

Study population

Adult patients aged 18 years or older with a diagnosis of DKA who were referred to the Emergency Department were enrolled in this study. The American Diabetes Association (ADA) [3] defines DKA as having the triad of hyperglycemia (blood glucose >250 mg/dL), a venous or arterial pH <7.3, and a serum bicarbonate <18 mmol/L, as well as the presence of positive serum or urine ketones.

A total of 51 participants were included in the study using a convenience sampling method. Patients who met the inclusion criteria were recruited consecutively as they presented during the study period. Individuals were excluded if they were pregnant, had end-stage renal disease, were undergoing dialysis, had a chronic systemic disease, or declined to provide written informed consent.

Sample size calculation

The sample size was calculated using the mean and standard deviation of serum potassium (4.49 ± 0.62 mmol/L) reported in a previous study of Singh et al [2]. Assuming a 99% confidence level and a relative precision of 5%, the required minimum sample size was calculated using the standard formula:

\[ n = \frac{(Z_{1-\alpha/2})^2 \times S^2}{d^2}. \] Substituting the values: \[ n = \frac{(2.58)^2 \times (0.62)^2}{(0.05 \times 4.49)^2} = 51. \]

Thus, a final sample of 51 patients was determined to be adequate for achieving the study objectives.

Operational definitions

Electrolyte imbalance was defined based on standard laboratory reference values used at the institution. Hypokalemia was defined as serum potassium <3.5 mmol/L, and hyperkalemia as serum potassium >5.5 mmol/L. Hypomagnesemia was defined as serum magnesium <1.6 mg/dL, and hypermagnesemia as serum magnesium >2.4 mg/dL [11]. An anion gap >14 was considered elevated and indicative of high-anion-gap metabolic acidosis (HAGMA) [5]. Renal potassium wasting was defined as either a urine potassium to urine creatinine ratio greater than 1.5 or a FEK exceeding 9.4% in hypokalemia.

Data collection procedure

Demographic and clinical data were collected during patient presentation using a structured case record form. Data included age, sex, duration, and type of diabetes, history of vomiting, polyuria, polydipsia, altered sensorium, fever, and drug use. Clinical evaluation included measurement of vital signs such as blood pressure, pulse rate, respiratory rate, and temperature. Neurological status and hydration status were also assessed. All laboratory and ECG investigations were performed before initiation of intravenous fluids or insulin therapy to ensure accuracy of baseline metabolic values.

Laboratory methods

Venous blood and urine samples were collected immediately after admission and before therapeutic intervention. Serum potassium was measured using the indirect ion-selective electrode method, which provides a reliable and specific quantification of potassium ions. Serum magnesium was determined using the xylidyl blue colorimetric method, where magnesium forms a colored complex with xylidyl blue in an alkaline medium, measured spectrophotometrically. Both parameters were analyzed using automated biochemistry analyzers under strict internal quality control protocols.

Arterial blood gas analysis was conducted using a potentiometric method to assess arterial pH and bicarbonate. The anion gap was calculated using the following formula:

\[ \text{Anion gap} = [\text{Na}^+] - \left( [\text{Cl}^-] + [\text{HCO}_3^-] \right). \]

Anion gap values greater than 14 mmol/L were categorized as high according to the standard reference by Kraut and Madias [14] and interpreted in the context of metabolic acidosis due to DKA.

Urine samples were collected in sterile containers at the time of admission. Urine potassium and urine creatinine were measured using spectrophotometric methods. The FEK was calculated using the formula:

\[ \text{FEK} (\%) = \frac{[\text{Urine K}^+] \times [\text{Serum Creatinine}]}{[\text{Serum K}^+] \times [\text{Urine Creatinine}]} \times 100. \]

Additionally, the urine potassium-to-creatinine ratio was computed to support assessment of renal potassium handling. These indices were used to identify inappropriate renal potassium loss in hypokalemic patients [19]. All tests were performed in the National Accreditation Board for Testing and Calibration Laboratories-accredited central clinical laboratory of the institution following standardized operating procedures. External quality assurance protocols were in place to ensure accuracy and reproducibility.

Statistical analysis

Statistical analysis was performed using IBM Statistical Package for the Social Sciences Statistics version 25.0 (IBM Corp., Armonk, NY). Means ± standard deviations were used for continuous variables, and frequencies (percentages) were used for categorical variables. Chi-square test or Fisher’s exact test was used to compare the correlation between categorical variables. For continuous variables, differences between the groups were tested with independent t-tests and one-way analysis of variance (ANOVA). Correlation analysis between serum potassium and serum magnesium levels was performed using Pearson’s correlation coefficient. p < 0.05 was considered statistically significant.

## Results

The mean age was 66.45 ± 14.02 years (t = 4.92; p = 0.001). The study population included 31 (60.8%) female and 20 (39.2%) male patients (χ² = 2.45, p = 0.117). The most common age group was 60-69, with 16 (31.4%) patients (χ² = 5.15; p = 0.023). Diabetes was present in 46 (90.2%) patients (χ² = 25.78; p < 0.001), and 23 (45.1%) patients received insulin therapy (χ² = 0.78; p = 0.376) (Table 1).

**Table 1:** Baseline characteristics of DKA patients SD: standard deviation; DKA: diabetic ketoacidosis

Characteristics	Value	Statistical test value	p value
Age (mean ± SD) (in years)	66.45 ± 14.02	t = 4.92	0.001
Gender, n (%)			
Male	20 (39.2%)	χ² = 2.45	0.117
Female	31 (60.8%)		
Most common age group (60-69 years), n (%)	16 (31.4%)	χ² = 5.15	0.023
Known diabetics, n (%)	46 (90.2%)	χ² = 25.78	<0.001
Insulin users, n (%)	23 (45.1%)	χ² = 0.78	0.376

The mean blood glucose level was 413.39 ± 96.61 mg/dL (range: 250-650 mg/dL). Systolic blood pressure was 128.1 ± 23.9 mmHg, ranging from 80 to 180 mmHg, and diastolic blood pressure was 75.5 ± 12.7 mmHg, ranging from 50 to 100 mmHg. The mean arterial pH was 7.15 ± 0.17 (<7.0-7.3). The average respiratory rate was 22.73 ± 4.37 breaths/minute (range: 16-36). Serum potassium was 4.69 ± 1.02 mEq/L (range: 2.8-7.1), serum creatinine was 1.28 ± 0.57 mg/dL (range: 0.43-2.70), serum magnesium was 1.87 ± 0.28 mg/dL (range: 1.30-2.90), and the anion gap was 16.28 ± 3.41 mEq/L (range: 11.8-27.6) (Table 2).

**Table 2:** Biochemical profile at presentation SD: standard deviation

Parameters	Mean ± SD	Range
Blood glucose (mg/dL)	413.39 ± 96.61	250-650
Systolic blood pressure (mmHg)	128.06 ± 23.94	80-180
Diastolic blood pressure (mmHg)	75.47 ± 12.65	50-100
Arterial pH	7.15 ± 0.17	<7 to 7.3
Respiratory rate	22.73 ± 4.37	16-36
Serum potassium (mEq/L)	4.69 ± 1.02	2.8-7.1
Serum creatinine (mg/dL)	1.28 ± 0.57	0.43-2.70
Serum magnesium (mg/dL)	1.87 ± 0.28	1.30-2.90
Anion gap	16.28 ± 3.41	11.8-27.6

**Figure 1:**
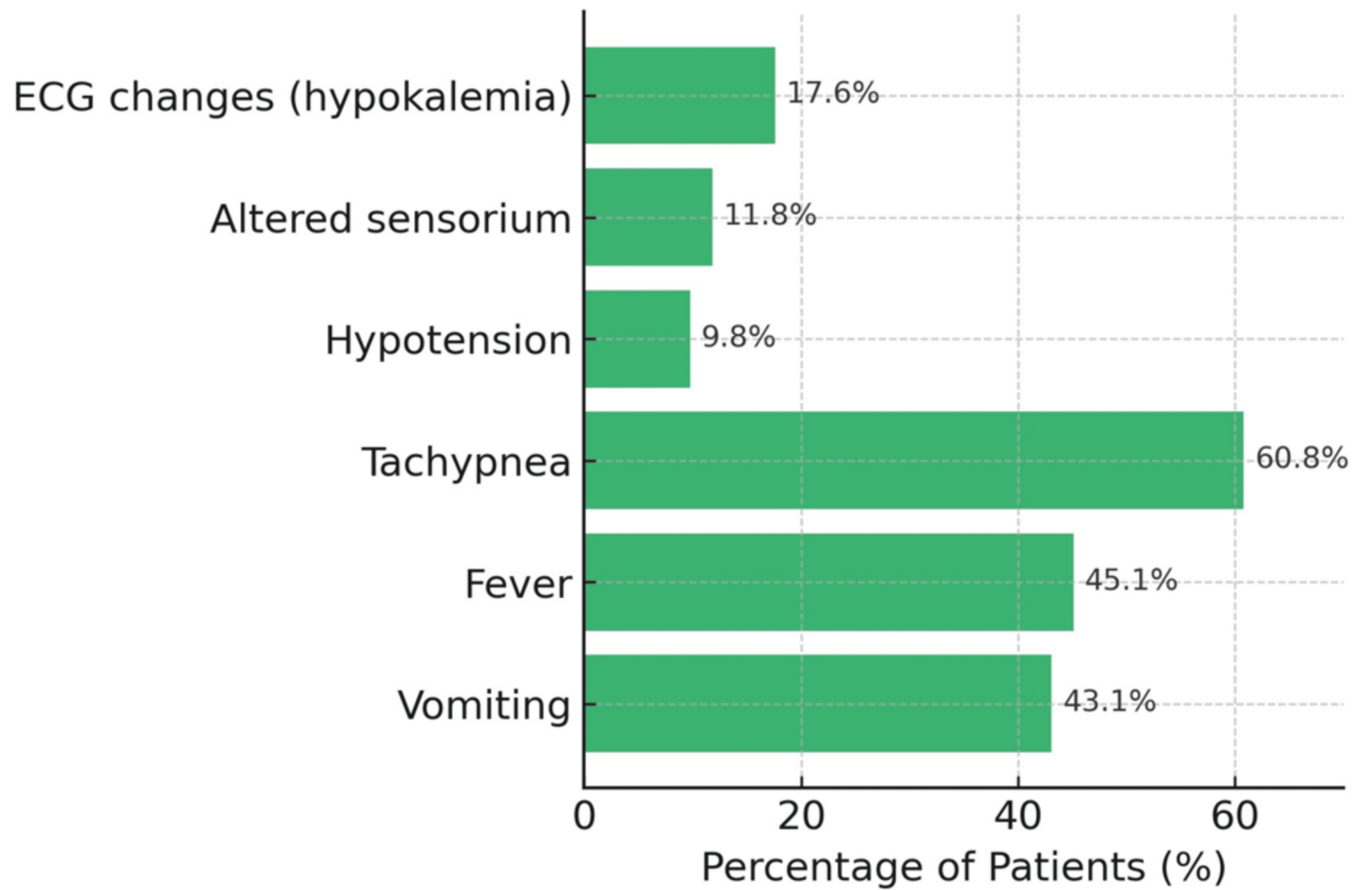
Clinical presentations

Tachypnea was the most common among clinical features, observed in 31 (60.8%) patients. Fever and vomiting occurred in 23 (45.1%) and 22 (43.1%) patients, respectively. ECG changes suggestive of hypokalemia were noted in 9 (17.6%) patients. Altered sensorium and hypotension were less frequent, seen in six (11.8%) and five (9.8%) patients, respectively (Figure 1).

An elevated anion gap (>14) was observed in 31 (60.8%) patients, with a mean value of 18.19 ± 3.09. A total of 19 (37.3%) patients had an anion gap in the range of 12-14, with a mean of 13.4 ± 0.54. Only 1 (2%) patient had a normal anion gap in the range of 10-12 mEq/L, with a value of 11.8. The difference in mean anion gap across these groups was statistically significant (F = 39.73, p < 0.001) (Table 3).

**Table 3:** Distribution of anion gap SD: standard deviation; ANOVA: analysis of variance

Anion gap range	n (%)	Mean AG ± SD	ANOVA (F)	p value
10-12 (mEq/L)	1 (2%)	11.8	39.73	<0.001
12-14 (mEq/L)	19 (37.3%)	13.4 ± 0.54		
>14 (mEq/L)	31 (60.8%)	18.19 ± 3.09		

Hypokalemia was observed in three (5.9%) patients: two had mild hypokalemia with a mean serum potassium level of 3.30 ± 0.14 mEq/L, and one had moderate hypokalemia with a serum potassium level of 2.80 mEq/L. These were the only patients evaluated for renal potassium loss using the FEK and the urine potassium/creatinine ratio. One-way ANOVA across potassium categories demonstrated a statistically significant difference in mean serum potassium levels (F = 66.75, p < 0.001), supporting the stratification of potassium status. In two mild cases of hypokalemia, urine creatinine was 157 mg/dL and urine potassium was 65 mmol/L, yielding a potassium/creatinine ratio of 0.41 mmol/mg and an FEK of 12.2%. In the single moderate hypokalemia case, urine creatinine was 180 mg/dL and urine potassium was 55 mmol/L, with a potassium/creatinine ratio of 0.31 mmol/mg and an FEK of 10.8%. These findings indicate renal potassium wasting in mild and moderate hypokalemia (Table 4).

**Table 4:** Evaluation of renal loss FEK: fractional excretion of potassium

Parameters	Degree of hypokalemia	
	Mild (n = 2)	Moderate (n = 1)
Urine creatinine (mg/dL)	157	180
Urine potassium (mmol/L)	65	55
K⁺/creatinine ratio (mmol/mg)	0.41	0.31
FEK (%)	12.2%	10.8%

**Figure 2:**
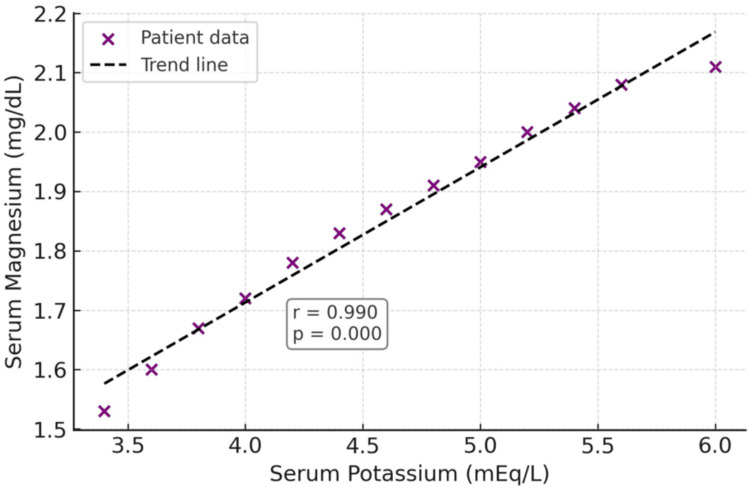
Scatter plot showing the correlation between serum potassium and serum magnesium levels in patients with diabetic ketoacidosis

A scatter plot demonstrated a strong positive linear correlation between serum potassium and serum magnesium in patients with DKA. The Pearson correlation coefficient was r = 0.982 with p < 0.001, indicating a strong association when assessing their relationship. The fitted trend line supported this linear relationship, suggesting that higher serum potassium levels are closely linked with elevated serum magnesium levels (Figure 2).

Among the 51 patients, normokalemia was the most common finding, observed in 34 (66.7%) cases. It was predominantly associated with moderate acidosis in 18 (72%) cases and mild acidosis in 14 (73.7%) cases. Hyperkalemia was present in 14 (27.5%) patients, mainly in association with severe acidosis in four (57.1%). Hypokalemia was the least frequent, occurring in three (5.9%) patients, with one case each in severe acidosis (14.3%), moderate acidosis (4%), and mild acidosis (5.3%) (Table 5).

**Table 5:** Severity of acidosis and distribution of serum potassium levels

Arterial pH range	Acidosis severity status	n (%)	Hypokalemia	Normokalemia	Hyperkalemia
<7.0	Severe	7	1 (14.3%)	2 (28.6%)	4 (57.1%)
7.0-7.2	Moderate	25	1 (4%)	18 (72%)	6 (24%)
>7.2	Mild	19	1 (5.3%)	14 (73.7%)	4 (21.1%)
Total		51 (100%)	3 (5.9%)	34 (66.7%)	14 (27.5%)

## Discussion

This study offers valuable insights into electrolyte disturbances and acid-base imbalances in DKA within an Indian population. The results corroborate and extend existing evidence, particularly concerning anion gap patterns, potassium homeostasis, and their correlations with arterial pH.

In this cohort, 31 (60.8%) patients presented with an elevated anion gap >14 mEq/L, indicating HAGMA. Meanwhile, 19 (37.3%) patients had a mildly elevated gap of 12-14 mEq/L, while one (2.0%) patient demonstrated a normal gap within the range of 10-12 mEq/L. These findings are consistent with previous studies that attribute HAGMA in DKA primarily to the accumulation of ketone bodies, including β-hydroxybutyrate and acetoacetate [3,20,21]. Among patients with more severe acidosis, underlying precipitating factors such as infection, missed insulin therapy, or other physiological stressors were clinically observed; however, a detailed subgroup analysis correlating these factors with the degree of acidosis was beyond the scope of the present study. The overall anion gap in this study, ranging from 11.8 to 27.6 mEq/L, suggests variability in ketone production or additional unmeasured anions, such as d-lactate [13,18]. The mean anion gap (16.28 ± 3.41) was slightly lower than values reported in Western cohorts (18-22 mEq/L) [12], possibly reflecting differences in population characteristics or metabolic responses.

Anion gap correlated strongly with acidosis severity (p < 0.001), supporting its role as a biomarker for DKA severity [14]. However, the persistence of HAGMA in some patients with milder acidosis (pH > 7.2) indicates possible contributions from other factors, such as hyperchloremia or renal dysfunction [22]. In addition, although renal potassium loss was observed in a subset of patients, hypokalemia in DKA is likely multifactorial. Factors such as reduced oral intake, gastrointestinal losses, transcellular potassium shifts, and variable renal perfusion states may all influence serum potassium levels, particularly in the context of dehydration and impaired renal function.

The mean arterial pH was 7.15 ± 0.17; severe acidosis (pH < 7.0) occurred in 13.7% of patients. These values meet the ADA diagnostic threshold (pH < 7.3) [7,23] but are slightly higher than those in some severe DKA reports (e.g., 7.10 ± 0.12) [24], possibly reflecting earlier presentation or compensatory respiratory alkalosis, as suggested by the elevated respiratory rate (22.73 ± 4.37 breaths/minute).

The severity distribution of acidosis was mild in 37.3%, moderate in 49%, and severe in 13.7%, mirroring trends from multiethnic populations [25]. Hyperkalemia predominated in severe acidosis (57.1%), likely due to acidosis-driven transcellular potassium shifts [26], whereas normokalemia dominated milder cases (72%-73.7%).

Overall, normokalemia (66.7%) was most common, followed by hyperkalemia (27.5%) and hypokalemia (5.9%). The low hypokalemia prevalence contrasts with reports of 10%-15% [20,27], possibly because delayed hospital presentations in other studies allowed renal potassium loss to develop. Potassium correlated strongly with magnesium (r = 0.982, p < 0.001), supporting their coregulation and the need for concurrent replacement [28]. The strong positive correlation observed between serum potassium and magnesium levels may be explained by their closely interrelated physiological regulation. Magnesium plays a critical role in maintaining intracellular potassium homeostasis by modulating Na⁺/K⁺-adenosine triphosphatase activity and reducing renal potassium wasting. Hypomagnesemia can impair cellular potassium uptake and promote renal potassium loss, thereby contributing to refractory hypokalemia. In the context of DKA, osmotic diuresis, insulin deficiency, and subsequent insulin therapy further influence the intracellular and extracellular distribution of both electrolytes. These findings highlight the importance of concurrent monitoring of magnesium levels in patients with DKA, even when serum potassium appears normal. Although routine supplementation in normokalemic patients cannot be universally recommended based on this study alone, early identification and correction of magnesium deficiency may help prevent subsequent potassium imbalance and improve overall electrolyte management.

Renal potassium wasting was evident in hypokalemic patients, with elevated FEK (10.8%-12.2%) and urine K⁺/creatinine ratios (0.31-0.41 mmol/mg), consistent with osmotic diuresis and hyperaldosteronism [29]. These findings highlight the importance of assessing renal potassium handling in DKA to guide replacement strategies [30].

The results reaffirm HAGMA as the predominant acid-base pattern in DKA and demonstrate that both anion gap and pH correlate strongly with acidosis severity. The observed potassium-magnesium relationship and evidence of renal potassium loss underscore the complexity of electrolyte management in DKA. Further research should quantify unmeasured anions and track dynamic electrolyte shifts during treatment to refine therapeutic protocols.

Limitations

This study has several limitations that should be acknowledged. First, the cross-sectional design limits the ability to establish causal relationships between electrolyte disturbances and the severity or progression of DKA. As the study was not longitudinal, dynamic changes in serum potassium and magnesium levels during treatment and recovery could not be assessed. Second, the sample size, particularly in the hypokalemic subgroup, was small, limiting the generalizability of findings on renal potassium loss. Third, unmeasured contributors to the anion gap, such as d-lactate and other organic acids, were not directly quantified, potentially underestimating their role in metabolic acidosis. Additionally, dietary intake, gastrointestinal losses, and detailed renal function dynamics were not systematically evaluated, which could affect electrolyte balance. Finally, clinical outcomes and temporal resolution of electrolyte abnormalities were not longitudinally tracked, limiting assessment of their impact on prognosis and treatment response.

## Conclusions

In conclusion, most patients with DKA in this study presented with normokalemia. However, clinically significant potassium abnormalities were also observed, including hyperkalemia and renal potassium loss in hypokalemic patients. A strong positive correlation between serum potassium and magnesium levels suggests interdependent regulation of these electrolytes during DKA. Anion gap analysis revealed elevated values in most cases, consistent with the predominance of HAGMA. These findings highlight the critical importance of comprehensive electrolyte monitoring, particularly concurrent assessment of potassium and magnesium, to ensure timely and effective correction during acute DKA management in emergency settings.
